# Identification of silibinin and isotretinoin as potent up-regulators of *sFRP4* (Wnt antagonist): *In silico* prediction and *in vitro* validation in breast cancer

**DOI:** 10.1371/journal.pone.0331735

**Published:** 2025-09-08

**Authors:** Rehana Ramzan, Shazia Anwer Bukhari, Azhar Rasul

**Affiliations:** 1 Department of Biochemistry, Government College University, Faisalabad, Pakistan; 2 Department of Zoology, Baba Guru Nanak University, Nankana Sahib, Pakistan; Cholistan University of Veterinary and Animal Sciences, PAKISTAN

## Abstract

Secreted frizzled-related protein 4 (*sFRP4*) plays a fundamental role in the regulation of Wnt signalling, which is crucial for cellular proliferation and differentiation. The *sFRP4* has garnered significant interest as a therapeutic target for metabolic diseases and cancer due to its mechanism of action. Although existing sFRP4 modulators show limited specificity and notable off-target effects, our study explores the potential of known bioactive compounds as more selective and less toxic alternatives. This study is based on the analysis of expression profiles, which demonstrated that the *sFRP4* gene exhibits aberrant expression in multiple cancers, including breast cancer. The protein’s primary involvement in cancer signaling pathways was determined through pathway enrichment analysis. The study employed molecular docking analyses and MD simulations to identify breast cancer-fighting small molecules with docking energies of less than −6 kcal/mol, targeting the sFRP4 binding hotspot using 100 natural or synthetic small molecules. Out of 100 screened compounds, Silibinin and Isotretinoin were selected based on docking results and further validated in vitro. In vitro investigations were carried out using the colorimetric MTT assay to assess cell viability and cytotoxicity based on metabolic activity. The potential of Silibinin and isotretinoin to upregulate the tumour suppressor *sFRP4* was further examined using ELISA and real-time quantitative PCR. Our study identified potential compounds for high-potential drug candidates against *sFRP4*, demonstrating their effectiveness in cancer cell death and upregulating *sFRP4* expression through improved drug design methods and experimental studies. In conclusion, our in-silico findings could facilitate the discovery of potential therapeutic agents against breast cancer. Silibinin and Isotretinoin impede cancer cell development in vitro; nonetheless, this study demonstrated that they directly upregulate *sFRP4* and induce apoptosis in breast cancer cells.

## Introduction

Breast cancer (BC) is one of the most common cancers worldwide, and its prevalence has been growing for decades. BC was the second most prevalent and fatal disease worldwide in 2022, contributing to 11.6% of new cases and 6.9% of cancer deaths. It was the most frequent cancer in women, contributing to one-sixth of global female cancer fatalities and one-fourth of diagnoses. Although it predominantly affects women (>99% of cases), its occurrence in men is extremely rare. The advancement and evolution of breast cancer are contingent upon genetic anomalies and hormonal dysregulation [1]. Breast cancer is a major source of mortality for women in low- and middle-income nations, where its impact is especially pronounced among these populations [2,3].

Breast cancer incidence is highest following menopause. A number of pathophysiological variables contribute to the onset of breast cancer, including gene mutations, particularly in the BRCA1 and BRCA2 genes, inherited genetic predispositions, hormonal exposure (oestrogen and progesterone), dietary influences, environmental exposure to carcinogenic chemicals, and unhealthy lifestyle choices [4–6]. Potential therapeutic targets in malignancies and reproductive system disorders include a range of proteins, enzymes, vascular endothelial and epidermal growth factor receptor [7–10].

Secreted Frizzled-related protein 4 (*sFRP4*) modulates Wnt signalling extracellularly via binding to the Wnt ligand and frizzled receptor. Therefore, it works as a Wnt ligand antagonist, blocking receptor interaction. *sFRP4* is the largest *sFRP* protein, weighing 39.9 kDa and containing 346 amino acids [11]. In several malignancies, diminished or eliminated expression leads to increased Wnt/β-catenin signaling and excessive cell proliferation [12,13]. Wnt pathway activation is another consequence of *sFRP4* gene silencing or reduced protein production. The first exon of *sFRP4* is flanked by numerous CpG islands. One of the ways genes are silenced, leading to a susceptibility to cancer, is via hypermethylation [14,15]. Restoring *sFRP4* expression presents a favourable therapeutic approach for mitigating breast cancer progression.

Bioactive phytocompounds with antioxidant, anti-inflammatory, and anti-proliferative effects have recently gained attention as potential cancer treatments. These natural compounds provide potential benefits compared to traditional medicines by inducing cytotoxic effects on cancer cells while reducing harm to normal cells. Our strategy involves screening a library of such compounds, including well-documented flavonolignans and retinoids known for their general anticancer properties, to identify specific modulators of sFRP4 [16,17]. Additionally, it modulates key signalling pathways involved in tumour progression [16]. These phytochemicals may provide a synergistic approach in targeting triple-negative breast cancer (TNBC), offering a potentially effective therapeutic strategy.

The drug development process begins with bioinformatics analysis, focusing on identifying effective drugs for treating clinical diseases. This involves identifying biological targets, such as proteins, genes, and RNA, using techniques like molecular docking, ADMET profiling, and three-dimensional structural modelling [18]. Identifying new chemicals that can upregulate the *sFRP4* (Wnt antagonist) is crucial for the biological development of drugs. This work utilised the PubChem database as the essential resource for screening compounds for drug development within the biological system [19,20]. An effective upregulator of the *sFRP4* target was found in our work by combining molecular docking with MD simulation methods. From an initial virtual screening of 100 bioactive compounds retrieved from databases, Silibinin and Isotretinoin were identified as the most promising candidates based on their most favourable docking scores, positive ADMET properties, and literature-supported anticancer activity.

This research seeks to find an upregulator of *sFRP4* using *in-silico* and *in-vitro* methodologies. Screening, different databases utilising the *sFRP4* target protein, investigation of diverse in-silico methodologies for modulating the molecular pathways linked to the progression of breast cancer, and the molecule’s confirmation in in vitro experiments.

## Methods

This study combines *in silico* and *in vitro* approaches to identify potential upregulators of sFRP4 in breast cancer. Computational analyses included gene expression profiling, ADMET predictions, molecular docking, and MD simulations, while experimental validation involved MTT assay, wound healing, ELISA, and qPCR.

### In silico studies

#### Survival analysis and gene expression.

Breast cancer patients’ *sFRP4* expression levels were measured using the UALCAN portal (https://ualcan.path.uab.edu/). The target genes were explored using the Cancer Genome Atlas (TCGA) database, first for invasive breast cancer and then for numerous cancer types. The Kaplan-Meier survival study analysed cancer progression survival.

#### Gene set enrichment analysis.

The gene set enrichment analysis for s*FRP4* was performed using Enricher (https://maayanlab.cloud/Enrichr/). Enrichr serves as a primary resource for performing enrichment analysis of mammalian gene sets. Using databases like KEGG, GO, and Reactome, as well as others that pertain to illnesses, biological processes, and recognized functions, it compares the given gene to a library of annotated genes. This tool may help understand target gene pathways.

#### Protein structure prediction and validation.

As reported by Rehman et al. [21] that no complete experimentally resolved structure of human sFRP4 was available in the Protein Data Bank (PDB), so the 3D structure was predicted using SWISS-MODEL based on homology modelling principles [22]. The resulting model was refined using GalaxyRefine, and the quality of the refined structure was evaluated using multiple bioinformatics tools, including ProSA, PDBsum, ERRAT, and Verify 3D [23–25]. These validation tools confirmed the stereochemical quality, residue environment compatibility, and non-bonded interaction quality of the model, making it suitable for downstream molecular docking analysis. Protein 3D structure prediction and validation were performed using the approach proposed by Rehman et al [21]. The predicted structure was evaluated through multiple bioinformatics tools, ensuring its accuracy and stability. Molecular docking and dynamic simulations confirmed the fidelity of the simulated protein-ligand interactions [26].

#### Database preparation for bioactive conformation.

A focused compound library was developed, consisting of natural phytochemicals and plant-derived bioactive molecules with reported anticancer and chemopreventive activities. These compounds were selected based on a literature search using academic databases including PubChem, Google Scholar, and Science Direct [27,28]. The compounds were selected based on reported or computationally predicted anticancer activity. Additional filters included drug-likeness (Lipinski’s Rule of Five) and favourable ADMET properties to ensure pharmacological relevance and safety. The selected ligands were retrieved from PubChem in SDF format, converted into three-dimensional structures using Progenesis SDF Studio, and exported in PDB format for downstream docking analyses [29]. These energy-minimized ligands were then utilized as input files in molecular docking simulations performed with AutoDock Vina to evaluate their binding affinity with the sFRP4 protein target [30].

#### Biological properties analysis.

Using Lipinski’s rule of five and pharmacokinetic profiles, each of the top compounds’ drug-likeness was evaluated using an in-silico analytical method [31,32]. The most favourable results were evaluated for ADMET qualities utilising SwissADME [33], ADMET lab 3.0 [34], ProTox-II [35], pkCSM [36,37], and admetSAR [38].

#### Prediction of the active site and preparation of the target protein.

An active pocket of the sFRP4 protein was predicted and the detection tool DoGSiteScorer. After manually examining the six predicted active pockets, we finally chose and proceeded with Active Pocket 1 as described previously [21,39,40]. Before docking, PyRx facilitated the import of the protein’s energy-minimized PDB structure, its conversion to PDBQT format, and choosing it as the target macromolecule [41,42].

#### Molecular docking.

To dock the *sFRP4* protein with several phytochemical ligands, the Auto Dock Vina run function of PyRx’s virtual screening program was used [37,43]. AutoDock Vina evaluates the binding affinity of the ligand with the desired protein using an empirical scoring approach. This empirical scoring formula served as the basis for calculating binding affinities. PyRx now contains input PDB files for ligands and targets. The binding site of the protein that was identified by the DoGSiteScorer was determined by the grid box centre and size measurements. The grid box’s size was set at 25.08 Å, y = 19.02 Å, and z = 25.0 Å, while the center’s dimensions were set at x = 26.11, y = −9.14, and z = 57.33 [44]. PyRx begins docking using Auto Dock Vina, producing many poses of the ligand attaching to the protein. The binding affinities of these poses are used to rank them. PyRx gives ligand-receptor interaction docking binding energies in kcal/mol. For active medications, a potential binding dock energy of −6 kcal/mol is frequently regarded as a threshold.

#### Study of ligand interactions.

Each phytochemical produced distinct effects after docking. The position with the zero RMSD value and the highest negative binding energy was chosen [38]. Using BIOVIA Discovery Studio, the Autodock-generated protein-ligand interaction findings were examined and displayed [45,46], Snapshots were taken of the best interaction poses. The study utilized BIOVIA Discovery Studio to analyse binding profiles and orientations of proteins’ active sites, utilizing energy dynamics of ligands for interaction analysis [47]. The study identifies non-covalent interactions in ligand conformations, including hydrogen bonds and hydrophobic contacts, and selects compounds for further research based on hydrogen bonding docking value [48]. This information may facilitate the creation of more efficacious pharmaceuticals by comprehending their behaviour under varying settings [49].

#### Targeted molecular dynamics (TMD) simulations.

For simulation investigations, the Desmond/Maestro (2022.1 version) was utilized [50,51]. The Protein Preparation Wizard of the Maestro interface was used in order to modify and pre-process the receptor-ligand complexes. The system was also constructed using the System Builder tool. The docked complexes were solvated in an orthorhombic simulation box using the TIP3P explicit water model. Counter-ions were added to neutralize the system, and the setup was then subjected to energy minimization and equilibration prior to the production MD run.

The Desmond software’s interaction diagram feature was used to study RMSF, how ligands twist, and the interactions between proteins and ligands, with the RMSD calculated as shown.


$$\begin{array}{c} RMSDx=\sqrt{1/N\sum {\mathrm{\backslash nolimits}}_{i=1}^{N}\left\langle \left(r_{1}^{\prime }\left(t_{x}\right)\right)-{\left(r_{i}\left(t_{ref}\right)\right)}^{2}\right\rangle } \end{array}$$ (1)

where N is the number of atoms in the atom selection; t ref is the reference time, (typically the first frame is used as the reference and it is regarded as time t = 0); and r’ is the position of the selected atoms in frame x after superimposing on the reference frame, where frame x is recorded at time t x. The procedure is repeated for every frame in the simulation trajectory. All TMD simulations were performed in triplicate under identical conditions to ensure reproducibility and consistency of ligand-induced conformational behaviour.

The Root Mean Square Fluctuation (RMSF) is useful for characterizing local changes along the protein chain. The RMSF for residue i is:


$$\begin{array}{c} {RMSF}_{i}=\sqrt{1/T\sum {\mathrm{\backslash nolimits}}_{i=1}^{N}\left\langle (r_{1}^{\prime }\;(t))-(r_{i}\;(t_{ref}\;){)}^{2}\right\rangle } \end{array}$$ (2)

where T is the trajectory time over which the RMSF is calculated, t ref is the reference time, r i is the position of residue i; r’ is the position of atoms in residue i after superposition on the reference, and the angle brackets indicate that the average of the square distance is taken over the selection of atoms in the residue.

### Biological evaluation (in-vitro studies)

#### Cell culture.

Triple-negative breast cancer (TNBC) cell line MDA-MB-231 was acquired from the NIBGE Cell Culture Collection. Cells were grown in DMEM with 10% FBS, 1% Penicillin-Streptomycin (100 IU/mL penicillin and 100 µg/mL streptomycin), 2 mM L-glutamine, and 1% NEAAs. Cells were cultured in tissue culture flasks at 37°C in a humidified 5% CO₂ environment. The cells were subcultured at a 1:3 split ratio and treated with 1–2 ml of a 0.25% trypsin-EDTA solution every three to five days.

#### MTT cell proliferation assay.

We used the MTT assay to evaluate anticancer efficacy and ascertain cell viability. At a density of 0.01 x 10⁶ cells/mL, MDA-MB-231 cells were seeded into a 96-well plate with 100 µL supplied to every well. After an overnight incubation period, the medium used for cell growth was removed, and the anticancer medication Silibinin was dissolved suitably before being administered to the cells with immediate effect. The cells were subjected to treatment with Silibinin and isotretinoin throughout a broad spectrum of concentrations (6.25−200 µg/ml). After a treatment period of 72 hours, 10 microliters of MTT (12 mM) reagent, with a reference number of V13154 and a lot number of 1753469, was administered to each well. Life Technologies in Carlsbad, California, United States, manufactured the reagent. Any morphological changes in the cells were seen using an inverted phase contrast microscope (IMT-2; Olympus, Tokyo, Japan), before the MTT reagent was added. The cells spent four hours. After the incubation period, formazan crystals were dissolved by adding 100 µL of DMSO; thereafter, the culture medium was gently removed from every well. The absorbance of every sample at 570 nm was then measured using a Synergy H1 hybrid multi-mode microplate reader (BioTek Instruments Inc., Winooski, VT, USA). Cell viability was determined using the accompanying formula:


%[100×(Sample Absorption/Control Absorption)]

The IC50 of cells treated with Silibinin and isotretinoin was determined using Prism (Graphpad Software, Inc., San Diego, CA).

#### Wound healing assay.

Cells were cultured in six-well plates and maintained at 37°C until achieving 80% confluence. The cells were spread out in a single layer and then scraped using a 200 µL autoclaved pipette tip. We shall rinse the cells with PBS after removing the medium. New DMEM with the specified amounts of Silibinin and isotretinoin was introduced to the cells for 0 and 24 hours. A microscope was used to examine and photograph the scraped wound (Olympus IMT-2 Inverted Microscope).

#### Quantification of *sFRP4* in the supernatants by ELISA..

The concentrations of *sFRP4* were quantified with ELISA kits from Glory Science Co., Ltd (GSCIENCE, CATALOG#: G9233, China, Hong Kong) per the manufacturer’s instructions.

#### Quantitative real-time polymerase chain reaction (qPCR).

Under a density of 2 x 105 cells/mL, T25 ultra-low flasks housed MDA-MB-231 cells. To extract RNA, the TRI reagent (Catalog No. 155960, Thermo, USA) was applied to the cells following 48 hours of treatment in DMEM. Following the manufacturer’s procedure, cDNA from the extracted RNA was synthesised using the SuperScript III first-strand cDNA synthesis kit (Cat No. 18080051, Life Technologies). Maxima SYBR Green/ROX qPCR Master Mix (2X) was used to amplify the cDNA in a real-time PCR cycler. We used the delta-delta CT technique to quantify the expression after normalizing the data to GAPDH. The data was analysed using GraphPad Prism software. Table 1 presents the entire sequences of the primers utilised by [52].

**Table 1:** The list of primers used to study the expression profile of the indicated gene.

Name	Forward primer	Reverse primer	Base pair	Accession No.
*GAPDH*	5’-GGAGTCCCCATCCCAACTCA −3	5’-GCCCATAACCCCCACAACAC −3’	173	XM_063276786.1
*sFRP-4*	5’AGGCAATAGTCACTGACCTTCC- 3’	5’CCTTTTTGCACTTGCACCGAT-3’	129	XM_017592435.1

### Statistical analyses

The statistical analyses were carried out in GraphPad Prism 8.0 using a One-way ANOVA. The mean ± standard deviation is used to communicate the results. The p-value was taken to be significant when it was ≤ 0.05.

## Results

### Aberrant expression of *sFRP4* in various cancers, including TNBC

**Fig 1:**
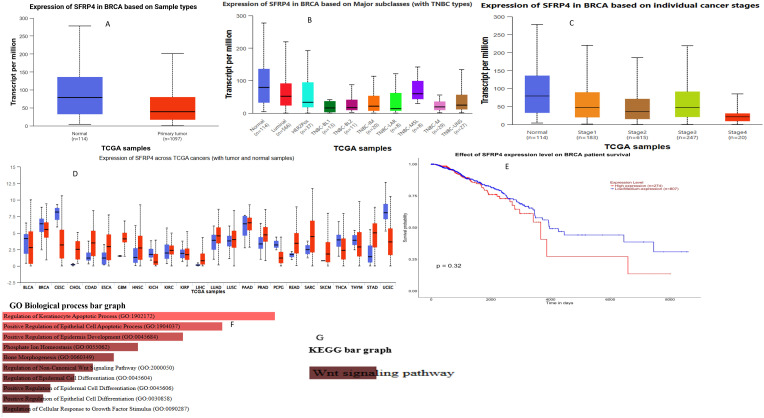
Expression of SFRP4 in A) Breast invasive carcinoma B) Main subtypes of breast cancer C) Individual D) with tumour and normal samples and, E) The Kaplan-Meier survival analysis and enrichment analysis of SFRP4 with, F) GO and G) KEGG biological process graph.

Transcripts per million (TPM) scores were used to quantify mRNA levels in tumorous cells compared to normal cells, allowing us to estimate the expression level of these putative target proteins. Access to clinical patient data is made possible via the UALCAN gateway by use of the TCGA database. The investigation started with the assessment of *sFRP4* expression levels in BC patients relative to normal breast tissue. According to Fig 1A, *sFRP4* expression is much higher in healthy individuals compared to those with invasive breast cancer. Compared to normal breast tissue, initial tumours had a much lower median expression of *sFRP4*. This suggests that *sFRP4* is downregulated in BC compared to normal tissue. Since sFRP4 is a known antagonist of the Wnt signalling pathway, its reduced expression in tumours suggests that loss of *sFRP4* may contribute to Wnt pathway activation, which is linked to cancer progression.

Comparative analysis of *sFRP4* expression across various breast cancer subclasses revealed significantly higher levels in healthy volunteers compared to patients with TNBC and HER2 receptor-positive BC, as shown in Fig 1B. Fig 1C demonstrates that *sFRP4* expression is stable during the first three stages of breast cancer, followed by a significant decrease in stage 4. As shown in Fig 1E, Kaplan–Meier survival analysis did not reveal a statistically significant difference in overall survival between patients with high and low/medium sFRP4 expression levels (p = 0.32). Fig 1F and G show GO and KEGG biological processes, respectively. Although sFRP4 is typically downregulated in breast cancer and associated with tumor suppression through Wnt signaling inhibition, this finding suggests that its prognostic value in survival may be limited or influenced by tumor subtype, stage, or additional regulatory mechanisms [53–58]. To confirm the results obtained from the clinical cancer database, we examined the levels of *sFRP4* gene expression in MDA-MB-231 BC cell lines using ELISA and qPCR.

To determine which biochemical pathways were more abundant in this set of genes, the Enricher server was used, as shown in the picture. The gene set primarily governs non-canonical Wnt signaling and participates in the apoptotic process. An effective method for blocking the Wnt signalling pathways’ beta-catenin compensation mechanism is to target *sFRP4* in breast cancer. In the analysis of TNBC cell lines, *sFRP4* expression was found to be slightly increased in MDA-MB-231, prompting the selection of this cell line for subsequent signalling investigations.

### In-silico findings

Computational analyses such as molecular docking and ADMET profiling have produced favourable results. The complex underwent a 100-nanosecond molecular dynamics (MD) simulation in a water-based environment, and the resulting deviations, interaction graphs, and fluctuations were examined.

### 3D-structure modeling and validation

**Fig 2:**
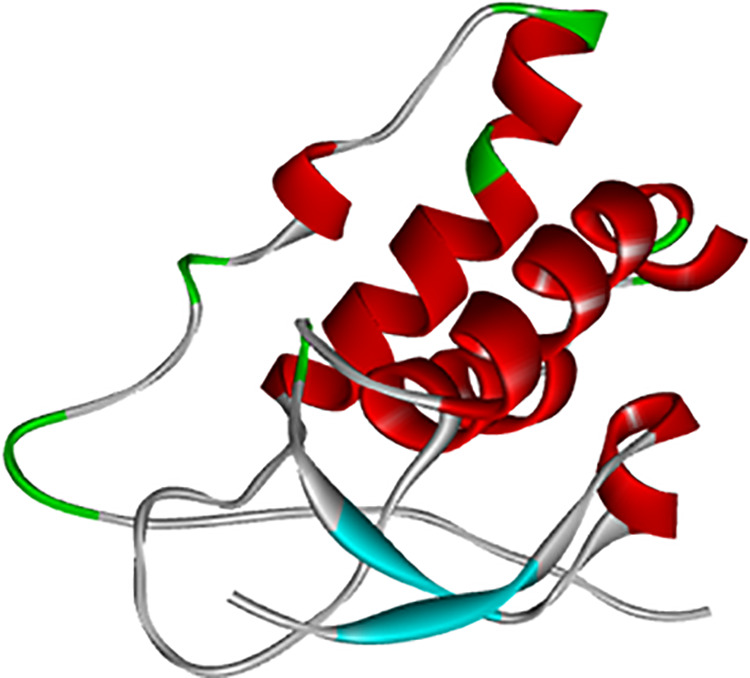
3D structure of the SFRP4.

The structural analysis of the *sFRP4* was extensively performed in the study of Rehman et al. [21], focusing on its molecular composition, active sites, and stability. Active site residues, such as Glu60, Tyr61, Glu63, Leu64, and His117, were identified as critical for ligand interactions through *in-silico* docking, as in Fig 2.

### Principal and molecular features of the ligand

Several potential therapeutic agents do not succeed in clinical trials because of their unsuitable ADMET properties. To evaluate the expected drug-likeness, ADME calculation is useful. Table 2 displays the aggregated ADMET endpoints. It is possible to deduce the molecular characteristics that are associated with drug-likeness by using five descriptors, which are as follows: the molecular weight (MW) must be ≤ 500 Da, the QPlogPo/w value must be five, the hydrogen acceptor must be ≤ 10, the hydrogen donor must be ≤ 10, and the Topological Polar Surface Area (TPSA) must be < 5. The standards follow the rule of five proposed by Lipinski. Most of the twenty-two parameters that make up the pharmacokinetic profile are within the acceptable range. The expected number of metabolic processes is within the permitted range, and the numbers are in line with the selected active ligand. The *sFRP4* protein molecular dynamics simulation and docked structures have been thoroughly examined for many factors. The chemical Silibinin and isotretinoin ADMET profile complies with Lipinski’s rule of five, and the binding affinity.

**Table 2:** Evaluation of the two most effective drugs using the parameters supplied by the computational prediction approach for drug likeness.

Ligand	Parameter	Silibinin	Isotretinoin
**Drug likeness**	Molecular Weight (<500 Dalton) (TPSA) (Å) 2 (<140) Heavy atom count (n atoms) H-bond donors (nOHNH) (≤5) H-bond acceptors (nON) (≤10) No. of rotatable bonds (≤10) LogS cLogP	482.12 155.1 4 35 5 10 4 −4.052 2.79	300.44 37.30 22 1 2 5 −3.905 4.186
**Adsorption**	Water solubility Caco-2 Permeability P glycoprotein inhibitor Intestinal absorption (% absorbed) Skin Permeability	−3.204 0.435 yes 61.861 −2.735	−4.924 1.61 No 94.419 −2.703
**Distribution**	BBB permeability (Log BB) CNS permeability (Log PS)	No −3.639	0.236 −2.114
**Metabolism**	CYP2D6 Substrate CYP3A4 Substrate CYP1A2 Inhibitor C YP2C19Inhibitor CYP2C9 Inhibitor	No Yes NO No No	No Yes No Yes Yes
**Excretion**	Total Clearance (Log ml/min/kg) Renal OCT2 substrate	−0.103 No	1.443 No
**Toxicity**	AMES toxicity Skin Sensitization Oral Rat Acute Toxicity (LD50) (mol/kg)	No No 2.559	No Yes 1.767

The chosen ligands were all inert across the blood-brain barrier, had excellent intestinal solubility, and positive CaCO2 permeability. Having shown little inhibitory potential against CYP1A2 and CYP2C19, the ligands were also beneficial towards cytochrome P450 enzymes. All of the lead ligands had poor total and renal clearance. Lastly, the AMES toxicity test was successful for the lead ligands that were chosen based on their toxicity prediction findings. The lead ligand ADMET drug tests did, however, provide satisfactory findings, suggesting that these leads may be good candidates for treatment against sFRP4.

### Protein-ligand interaction analysis

Of the 100 bioactive compounds analysed, merely three compounds (Silibinin, Isotretinoin, and Aristolochic acid) displayed binding energies lower than −6 kcal/mol, as shown in Table 3.

**Table 3:** Molecular docking energy values of SFRP4 with candidate compounds.

Sr. No	Ligand Name	Molecular Formula	Binding Score (Kcal/mol)	Interacting Residues in the Active Pocket
**1**	Silibinin	C25H22O10	**−7.5**	Met112,Pro120,Leu64, Glu63 and Gln60
**2**	Isotretoinin	C20H28O2	**−6.2**	Val67,Tyr115,Leu64, His 117,Met112, Tyr61
**3**	Aristolochic acid(2236)	C17H11NO7	**−6**	Gln60,Pro120,Ty61Met112
**4**	Isorhamnetin	C16H12O7	**−5.9**	Leu64,Tyr61,Pro120
**5**	Lupeol	C30H50O	**−5.9**	Tyr61,Leu64,His117,Pro120,His48,Met112
**6**	Stigmasterol	C29H48O	**−5.8**	Tyr61,Pro120,Met112,Leu64
**7**	Caffeic Acid	C9H8O4	**−5.5**	His117,Glu63
**8**	Isoeugenol	C10H12O2	**−5**	Met112,Leu64

**Fig 3:**
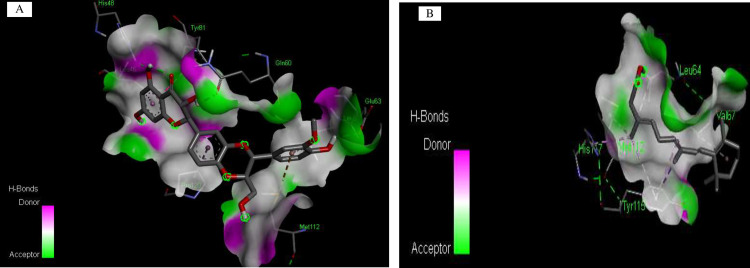
Predicted binding conformation of Silibinin and Isotretinoin with the designated ligand-binding site of sFRP4. A, B Interfaces are produced through the participation of protein-ligand complexes.

Among the three compounds analysed, only Silibinin demonstrated binding affinity to the designated receptor binding site, establishing robust polar interactions with three critical residues, specifically Glu63 and Gln60. Furthermore, it was demonstrated that Silibinin established highly stable interactions with critical residues, specifically Glu63 and Gln60 within the hydrophobic cleft of the target site as in Fig 3A, whereas the ligand isotretinoin exhibited three types of interfaces Pi-Pi stacking, Pi-sigma, and Pi-alkyl with amino acids Tyr61, Tyr115, His117, Val, Met112, and Leu64 on the target protein as shown in Fig 3B.

### Modelling the protein-ligand interaction

The docked structure underwent simulation for a duration of 100 ns following the equilibration phase, during which multiple metrics were plotted to demonstrate its stability. Trajectories were extensively analysed, with RMSD, RMSF, and intermolecular interactions identified throughout the simulation.

### Root mean square deviation and root mean square fluctuations

**Fig 4:**
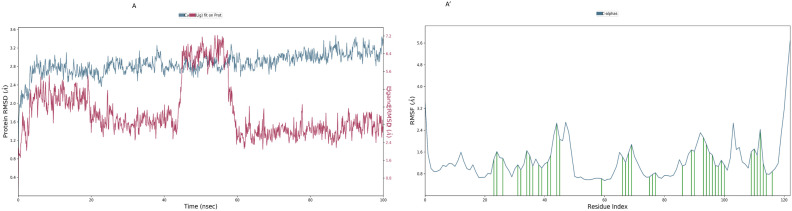
MD. Simulation results in A, A՛: Protein RMSD and RMSF for Silibinin complex.

RMSD is utilized to quantify exact deviations throughout the simulation time, and our research investigated their entire courses. Fig 4A, A՛ illustrates the RMSD for Silibinin complex, and Fig 4B, B՛ indicates the isotretinoin complex, and the secreted protein *sFRP4*, which is the target protein, ranges from 0 to 100 nanoseconds.

The protein in the Silibinin protein complex exhibited an initial variation of 2.5 Å, and the ligand a deviation of 16 Å between 0 and 10 nanoseconds.

Fig 4A illustrates the dynamic stability of both the protein (Cα atoms) and the ligand over a 100-nanosecond molecular dynamics simulation. The protein RMSD (blue line) shows an initial rise during the first 10 ns, stabilizing around 2.8–3.0 Å for the remainder of the simulation, indicating that the protein maintains a relatively stable conformation throughout. In contrast, the ligand RMSD (maroon line) exhibits more fluctuation. Initially, the ligand RMSD increases and fluctuates between 2.0–3.0 Å up to around 45 ns, suggesting moderate conformational flexibility within the binding pocket. However, a sharp spike is observed between approximately 45–58 ns, reaching up to ~7.2 Å, which may indicate a significant conformational change or partial unbinding event. After this spike, the ligand RMSD drops and stabilizes around 3.0 Å, suggesting that the ligand rebinds or adopts a new stable conformation. Overall, while the protein remains structurally stable, the ligand undergoes transient instability, possibly reflecting flexible binding dynamics or alternate binding modes within the active site. This conformational event was consistently observed across all three replicate simulations, confirming it is a genuine feature of the ligand’s dynamics rather than a simulation artifact.

Based on the RMSF analysis of Silibinin complex Fig 4A՛ shown in the graph, most of the Cα residues display fluctuations below 3.0 Å, indicating that the protein maintains overall structural stability throughout the simulation. However, elevated flexibility is observed at specific regions, particularly near the C-terminal end (e.g., around residue 120), where the RMSF reaches approximately 5.6 Å. This localized mobility may reflect solvent-exposed loops or terminal regions with dynamic roles in the protein’s function. Notably, residues such as GLN60 and GLU63 exhibit moderate fluctuations yet are critical in maintaining the stability of the protein-ligand complex, as they are involved in hydrogen bonding interactions with the ligand. Additionally, hydrophobic contacts between the ligand and residues Met112, Pro120, and Leu64 suggest key stabilizing interactions at the binding interface. These findings underscore the functional importance of these residues in ligand recognition and binding, highlighting them as potential targets in structure-based drug design. The combination of stable core residues and flexible interaction regions provides valuable insight into the dynamic behaviour of the protein during molecular recognition events.

**Fig 5:**
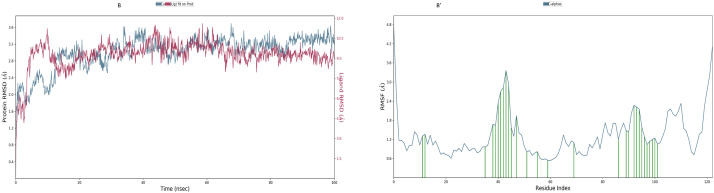
MD. Simulation results in (B, B՛): Protein RMSD and RMSF for Isotretinoin complex.

Fig 5B shows the RMSD values for both the protein and the ligand over a 100 ns period. Initially, at 0 ns, both RMSD values are low, indicating stability in both structures. As the simulation progresses, the protein experiences larger fluctuations in RMSD, but the values remain below 3 Å, suggesting it maintains its overall structure with only minor conformational changes. The ligand’s RMSD fluctuates less and stays under 3 Å throughout, indicating that it remains in a stable conformation within the protein’s binding site. Both structures stabilize around 90–100 ns, with minimal deviation. The protein’s RMSD values are slightly higher, showing more flexibility, while the ligand’s lower RMSD signifies its stable binding.

Our analysis of the Cα and ligand contacts for the RMSF in Fig 5B՛ revealed that the ligand isotretinoin interacts hydrophobically with Met112 and Leu64. Hydrophobic contacts are generated at Tyr61, Tyr115, His117, Val, Met112, and Leu64 amino acids (active site) on the protein by the ligand isotretinoin. Overall, most of the protein remains stable with RMSF values below 3.0 Å, indicating secure structural integrity during the simulation. The localized flexibility in specific regions may contribute to the protein’s dynamic behaviour or interactions, making these areas of interest for further investigation.

### Simulation interaction analysis

**Fig 6:**
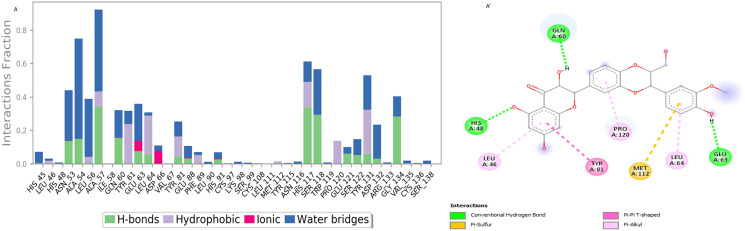
Simulation interaction diagram of silibinin (A, A՛) 2-D interactions illustration of the protein–Ligand.

Stable receptor-ligand interactions rely on the interaction strengths between the ligand and the target protein, *sFRP4*, making it essential to identify the amino acid residues that engage with the ligands via molecular dynamics simulations. Fig 6A, A՛ highlights that Silibinin interacts with its protein target through a combination of hydrogen bonding, hydrophobic interactions, and water-mediated bridges, primarily involving residues GLN60, GLU63, LEU46, PRO120, and MET112. These interactions contribute significantly to the stability and binding affinity of the Silibinin protein complex during molecular dynamics simulations.

**Fig 7:**
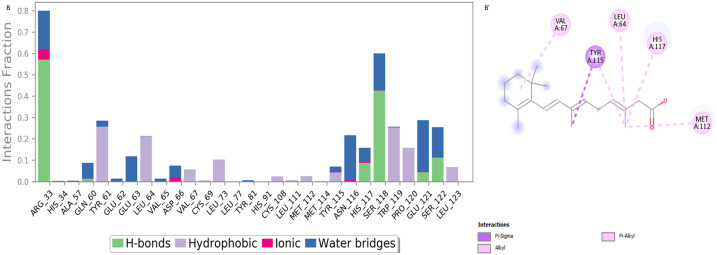
Simulation interaction diagram of isotretinoin (B, B՛) 2-D interactions illustration of the protein–Ligand.

Fig 7B, B՛ shows that the ligand primarily interacts with the protein through hydrophobic interactions (e.g., with MET112, TYR115, VAL67, and LEU46) and is stabilized by water-mediated bridges. The interaction diagram shows 2D interaction maps of docked complexes based on bioactive compounds, which illustrate how interactions are preserved over the simulation trajectory. Compounds Silibinin and Isotretinoin were similar to the co-crystal ligand’s acceptor and donor hydrogen bond characteristics. Significant interactions between Silibinin’s hydrogen bonds were highlighted by the counts of donor and acceptor bonds. The compounds Silibinin and Isotretinoin were shown in 2D interaction maps as having hydrogen bonds, pi-pi T-shaped, alkyl interactions, pi-sigma interactions, and pi-alkyl interactions, respectively.

These interactions illustrate how high-energy aromatic amino acid constituents are expected to be involved in organizing the adenine ring within the protein during its production. In addition, electrostatic interactions were observed between the various components of the amino acid composition. Figs 6 and 7 illustrate the quantity of unique intermolecular interactions established by all pocket residues with the ligand-binding area. The compounds isotretinoin and Silibinin were successfully docked and verified for higher-quality docking outcomes. Certain residues showed comparable hydrophobic and hydrogen bonding properties to those of amino acids.

### In-vitro study

#### Anti-proliferative activity in MDA-MB-231 (TNBC) cell.

**Fig 8:**
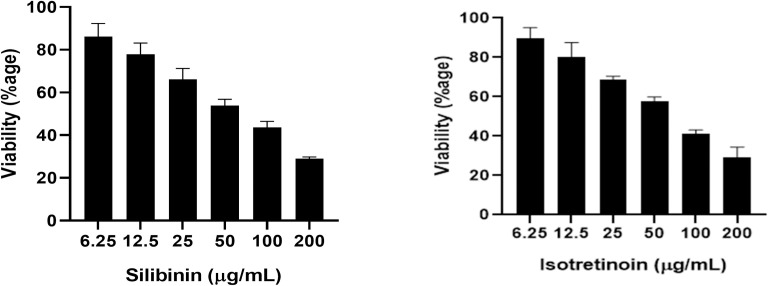
Anti-proliferative activity of Silibinin and isotretinoin in a dose-dependent manner by MTT assay.

Silibinin and Isotretinoin, identified as promising compound candidates through in silico results, were tested for their toxicity on MDA-MB-231 (TNBC) cells using the MTT method. As shown in Fig 8, Silibinin and Isotretinoin reduced the cell viability in a time- and dose-dependent manner. The results suggest that both compounds were not toxic for normal cells, showing their specific toxicity against the breast cancer cell line. Hence, Silibinin and Isotretinoin suppress cell growth and proliferation of MDA-MB-231 cells.

### Silibinin and isotretinoin inhibit the migration of MDA-MB-231 cells

**Fig 9:**
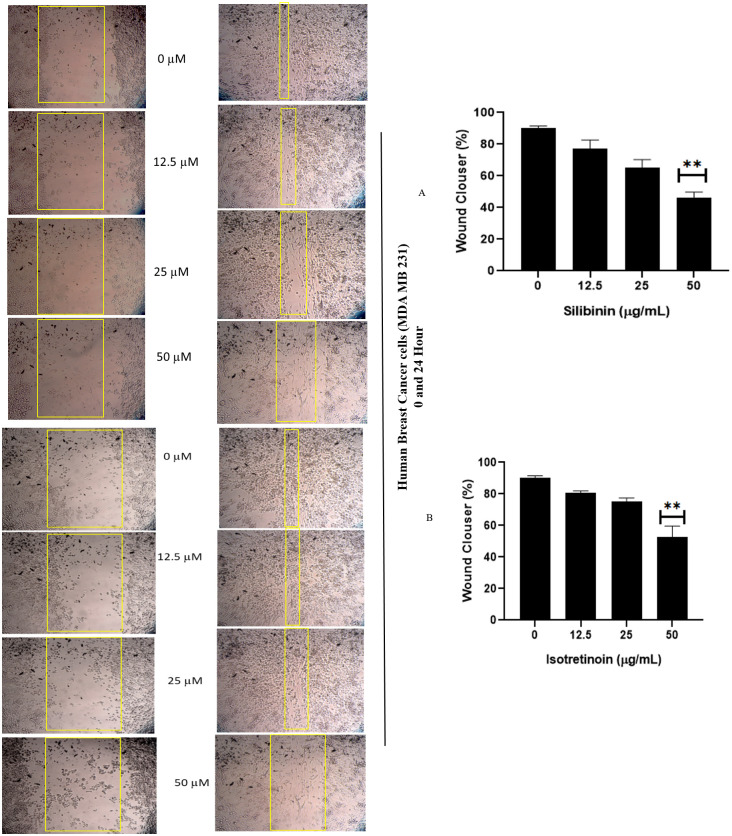
Silibinin and isotretinoin (A, B) stop MDA-MB-231 cells from migrating. Means ± SD of three separate experiments provide the data reported in A and B. **P < 0.01 in comparison to the control cohort.

Since Silibinin has previously demonstrated the ability to suppress invasion in different cancer cell lines with other targets [59–62]. Previous studies have demonstrated that 13CRA (isotretinoin) can impede the multiplication of cancer cells, namely those associated with the stomach and breast [63]. We tested whether Silibinin and Isotretinoin could also exert anti-metastatic effects in breast cancer cells. This experiment used an in vitro wound healing test to estimate the Silibinin effects and Isotretinoin on migrating MDA-MB-231 cells. The number of cells that migrated into the scratched region was determined by taking pictures after treatment began, and then the percentage of migration was calculated from those numbers. Fig 9A and B illustrates the outcomes of the migratory capacity assessment conducted on MDA-MB-231 cells by a wound-healing assay.

According to Fig 9A, B, during a 24-hour incubation period for injured cells, the groups treated with Silibinin and Isotretinoin showed an increase in percentage wound width at 12, 25, and 50 µg/ml, respectively. According to the study’s findings, it considerably and independently decreased the migration of triple-negative breast cancer cells MDA-MB-231.

### Expression analysis of *sFRP4*

**Fig 10:**
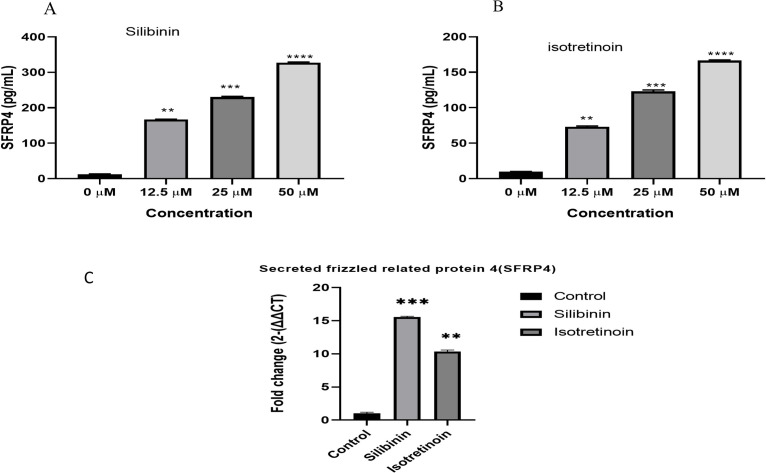
The expression of sFRP4 in TNBC MDA-MB-231 cell lines after treatment with silibinin and isotretinoin and its subsequent effects. The cells were subjected to a range of doses of silibinin and isotretinoin, ranging from 12.5 to 50 μM, for 48 hours. The findings of these investigations are presented as the means ± standard error of the mean (SEM) from three separate experiments. Statistical significance was assessed in Figures A and B. Effect of Silibinin and Isotretinoin affects the mRNA expression of the Wnt/β-catenin antagonist sFRP4 following 48 hours of exposure. Comparatively to untreated controls, both compounds affect the expression of sFRP4 gene in Figure C (**P < 0.05, ***P < 0.001).

Based on the ELISA results, both compounds exhibited remarkable inhibition of cell proliferation by blocking the binding of Wnt protein in the Wnt signalling pathway, and *sFRP4* expression was upregulated. In contrast, beta-catenin signalling overactivation significantly reduced *sFRP4* expression in breast cancer cells Fig 10A, B. Cells were harvested in order to use ELISA to measure the expression levels of *sFRP4*.

### Quantitative real-time polymerase chain reaction (qPCR)

Real-time qPCR was employed to evaluate the influence of both medications on the expression of Wnt/β-catenin target genes, namely the *sFRP4* tumour suppressor gene, following 24 hours of exposure to MDA-MB-231. A notable downregulation of Wnt gene expression and an upregulation of the *sFRP4* tumour suppressor gene were detected in a dose-dependent manner in Fig 10C.

## Discussion

Breast cancer development, metastatic dispersion, stemness, and resistance to multiple drugs have been linked to a complicated interaction of numerous signalling pathways that restrict the patient’s overall survival and effective treatment targeting [64]. Bioinformatics analysis revealed that *sFRP4* is a viable target for TNBC treatment. Gene expression research indicated aberrant *sFRP4* expression in a diversity of malignancies, including breast cancer. The possible functions of the main signalling pathways involving *sFRP4* in cancer development were further highlighted by the pathway enrichment analysis. Furthermore, docking investigations of *sFRP4* with various drugs and inhibitors have shown that Silibinin and Isotretinoin have the highest binding energy, making it the most promising choice.

The binding energies of the repurposed drugs isotretinoin and Silibinin with *sFRP4* were greater than those of the other compounds. It is necessary to recognize the intrinsic drawbacks of molecular docking, including its dependence on static structures and the possibility of inaccurate predictions of dynamic protein-ligand interactions. To overcome these limitations and improve the stability of the results, molecular dynamics (MD) simulations were used. These further investigations confirmed the potential effectiveness of Silibinin and Isotretinoin in contrast to the control inhibitors by shedding light on their binding stability with the target proteins. According to the findings of the MD simulation, Silibinin had a stronger and more consistent interaction with *sFRP4*. It is shown that Silibinin has stronger electrostatic and van der Waals forces. It was stated that Silibinin and Isotretinoin had a robust interaction with the target proteins.

*In-vitro* experiments were used to confirm the in-silico predictions of Silibinin and Isotretinoin’s anticancer therapeutic potential. Cell lines MDA MB 231 were chosen for the in vitro investigation due to their resemblance to human-origin breast cancer [65]. With the tetrazolium-based MTT assay, the cytotoxic effects of isotretinoin and Silibinin on these cells were evaluated [66]. Previous studies indicate that compounds at elevated concentrations (150–250 μM) inhibit cell proliferation, leading to apoptosis in MDA-MB-231 cells [67,68]. The results demonstrate that the MDA-MB-231 cells’ ability to migrate and invade is inhibited at lower doses of the two bioactive compounds. These findings were determined to be consistent with other studies [63,67,69,70]. Silibinin and Isotretinoin were shown to have anticancer properties in vitro. Interestingly, in EMT-induced TNBC cells, the IC50 for both drugs was decreased. Isotretinoin and Silibinin increased oxidative stress and ROS production, and upregulated the Wnt antagonist *sFRP4*, according to the results.

Through the production of ROS, both potent hits mechanistically cause TNBC cells to undergo apoptosis, which results in cell death [69]. Furthermore, prior research has demonstrated that the activation of the Wnt/β-catenin signaling pathway occurs more often in TNBC and is associated with worse clinical outcomes [71,72]. The case for *sFRP4* as a breast cancer therapeutic target is becoming stronger by the day. Several investigations have shown that Silibinin has cytotoxic effects on breast cancer cells: these effects were shown [73–75].

To further our comprehension of how retinoids impede the progression of breast cancer cell lines, we investigated the effect of 13cRA on inducing apoptosis in the MDA-MB-231 cell line of breast cancer. Proliferation experiments provide dose-dependent evidence that 13cRA (isotretinoin) inhibits cell growth in MDA-MB-231 cell lines. Furthermore, we found that Silibinin can upregulate the expression of the *sFRP4* gene and suppress the Wnt/β-catenin signaling pathway in breast cancer cells. Furthermore, we discovered that the effects of Silibinin on the Wnt/β-catenin signalling pathway occurred at doses that were equivalent to those necessary to reduce the proliferation of breast cancer cells. Taken together these data indicate that 13-cis RA inhibit the cell growth and induces apoptosis in breast cancer cell line by a RAR-independent mechanism. The inhibitory effects of both drugs on MDA-MB-231 are associated with its anti-breast cancer properties.

Breast cancer cells (TNBC) are distinguished by their increased Wnt/beta-catenin activity, which promotes cell migration and results in an invasive phenotype. The overexpression of downstream genes, including Cyclin D1 and CD44, has been associated with the disruption of the Wnt/β-catenin signaling cascade [76–78]. The investigation of gene expression by RT-PCR shown that both drugs markedly suppressed the expression of genes associated with the beta-catenin cascade. Furthermore, both Silibinin and Isotretinoin were shown to significantly (P < 0.05) enhance the expression of the tumour suppressor gene *sFRP4*. Ultimately, the discovery of this signalling network that drives EMT development in TNBC paves the way for novel treatment approaches that precisely target the gene which is part of this signalling cascade.

## Conclusion

This investigation established Silibinin and Isotretinoin as promising therapeutic agents that effectively upregulate the Wnt antagonist *sFRP4* in TNBC. Molecular docking and dynamic simulations confirmed strong, stable interactions with *sFRP4*, particularly for Silibinin. In vitro assays revealed dose-dependent cytotoxicity, reduced migration, and significant upregulation of *sFRP4*, leading to apoptosis in MDA-MB-231 cells. These findings position *sFRP4* as a promising therapeutic target for TNBC. In-vivo pharmacodynamics and safety characteristics of these molecules should be investigated in further studies. Investigating combination therapies with other pathway inhibitors could enhance anticancer efficacy. Long-term studies on resistance mechanisms and bioavailability will further refine their clinical applicability.

## Supporting information

S1 File (DOCX)
