# Supplementary material for: Identification of silibinin and isotretinoin as potent up-regulators of sFRP4 (Wnt antagonist): In silico prediction and in vitro validation in breast cancer
Source: PLoS One. 2025 Sep 8;20(9):e0331735. doi: 10.1371/journal.pone.0331735 (PMC12416665; doi:10.1371/journal.pone.0331735)
Supplement: S1 File — (DOCX) [file pone.0331735.s001.docx]

**Supporting information**

**Result for Anti-proliferative activity of Silibinin in a dose-dependent manner by MTT assay**

| **Absorbance** | **1** | **2** | **3** | **%viability** | **%viability** | **%viability** | **Average** | **S.D** |
| --- | --- | --- | --- | --- | --- | --- | --- | --- |
| **Control** | **0.308** | **0.299** | **0.3035** | **100** | **100** | **100** | **100** | **0** |
| **200** | 0.087 | 0.089 | 0.088 | 28.24675 | 29.76589 | 28.99506 | 29.00257 | 0.759594 |
| **100** | 0.126 | 0.139 | 0.1325 | 40.90909 | 46.48829 | 43.65733 | 43.68491 | 2.789704 |
| **50** | 0.175 | 0.152 | 0.1635 | 56.81818 | 50.83612 | 53.8715 | 53.84193 | 2.99114 |
| **25** | 0.188 | 0.213 | 0.2005 | 61.03896 | 71.23746 | 66.0626 | 66.11301 | 5.099435 |
| **12.5** | 0.256 | 0.217 | 0.2365 | 83.11688 | 72.57525 | 77.92422 | 77.87212 | 5.271009 |
| **6.25** | 0.247 | 0.276 | 0.2615 | 80.19481 | 92.30769 | 86.16145 | 86.22132 | 6.056665 |

**Result for Anti-proliferative activity of isotretinoin in a dose-dependent manner by MTT assay**

| **Absorbance** | **1** | **2** | **3** | **%viability** | **%viability** | **%viability** | **Average** | **S.D** |
| --- | --- | --- | --- | --- | --- | --- | --- | --- |
| **Control** | **0.308** | **0.299** | **0.3035** | **100** | **100** | **100** | **100** | **0** |
| **200** | 0.106 | 0.073 | 0.0895 | 34.41558 | 24.41472 | 29.48929 | 29.43986 | 5.000618 |
| **100** | 0.121 | 0.129 | 0.125 | 39.28571 | 43.14381 | 41.18616 | 41.20523 | 1.92912 |
| **50** | 0.182 | 0.17 | 0.176 | 59.09091 | 56.85619 | 57.99012 | 57.97907 | 1.117402 |
| **25** | 0.217 | 0.201 | 0.209 | 70.45455 | 67.22408 | 68.86326 | 68.8473 | 1.615292 |
| **12.5** | 0.289 | 0.238 | 0.2635 | 93.83117 | 79.59866 | 86.82043 | 86.75009 | 7.116514 |
| **6.25** | 0.293 | 0.261 | 0.277 | 95.12987 | 87.29097 | 91.26853 | 91.22979 | 3.919594 |

**Result for Silibinin stops MDA-MB-231 cells from migrating. Means ± SD of three separate experiments provide the data reported in A and B. **P < 0.01 in comparison to the control cohort**

| **Conc. ug/ml** | **Wound Clouser (%)** | **SD** | |
| --- | --- | --- | --- |
| 0 | 90.06333 | | 1.489978 |
| 12.5 | 77.37 | | 5.321738 |
| 25 | 65.14333 | | 5.134481 |
| 50 | 46.21667 | | 3.583577 |

**Result for isotretinoin stops MDA-MB-231 cells from migrating. Means ± SD of three separate experiments provide the data reported in A and B. **P < 0.01 in comparison to the control cohort**

| **Conc. ug/ml** | **Wound Clouser (%)** | **SD** |
| --- | --- | --- |
| 0 | 90.06333 | 1.489978 |
| 12.5 | 80.70333 | 1.154701 |
| 25 | 75.47667 | 1.980311 |
| 50 | 52.88333 | 6.712893 |

**S10 Figure:** The expression of sFRP4 in TNBC MDA-MB-231 cell lines after treatment with silibinin and isotretinoin and its subsequent effects. The cells were subjected to a range of doses of silibinin and isotretinoin, ranging from 12.5 to 50 μM, for 48 hours. The findings of these investigations are presented as the means ± standard error of the mean (SEM) from three separate experiments. Statistical significance was assessed in Figures A, B, and C. The Effect of Silibinin and Isotretinoin affects the mRNA expression of the Wnt/β-catenin antagonist sFRP4 following 48 hours of exposure. Compared to untreated controls, both compounds affect the expression of the sFRP4 gene in Figure C (**P < 0.05, ***P<0.001).

**One-way ANOVA of silibinin**

| **Conc.** | **1** | **2** | **SFRP4(pg/ml)** |
| --- | --- | --- | --- |
| 0 µM | 10.4 | 13.4 | 12 |
| 12.5 µM | 166.2 | 167.8 | 167 |
| 25 µM | 232 | 228 | 230 |
| 50 µM | 329 | 325 | 327 |

**One-way ANOVA of Isotretinoin**

| **Conc.** | **1** | **2** | **SFRP4(pg/ml)** |
| --- | --- | --- | --- |
| 0 µM | 10.1 | 9.9 | 10 |
| 12.5 µM | 74.2 | 71.8 | 73 |
| 25 µM | 121 | 125 | 123 |
| 50 µM | 167.4 | 165.6 | 166.5 |

**Expression of genes was measured by qPCR**

|  | **CT values** | | | |  |
| --- | --- | --- | --- | --- | --- |
|  | **GAPDH** | | **SFRP-4** | | **SD** |
| **Control** | 25.6 | 25.29 | 28.92 | 29.18 | 0.183848 |
| **Silibinin** | 26.9 | 26.96 | 26.4 | 26.65 | 0.176777 |
| **Isotrenotinin** | 26.1 | 26.29 | 26.6 | 26.26 | 0.240416 |
|  | Mean CT value | |  |  |  |
|  | GAPDH | SFRP-4 |  |  |  |
| **Control** | 25.45 | 29.05 |  |  |  |
| **Silibinin** | 26.93 | 26.53 |  |  |  |
| **Isotrenotinin** | 26.20 | 26.43 |  |  |  |
|  | **ΔCT values** |  |  |  |  |
| **Control** | 3.605 |  |  |  |  |
| **Silibinin** | -0.405 |  |  |  |  |
| **Isotrenotinin** | 0.23 |  |  |  |  |
|  | **ΔΔCT values** |  |  |  |  |
| **Control** | 0 |  |  |  |  |
| **Silibinin** | -4.01 |  |  |  |  |
| **Isotrenotinin** | -3.37 |  |  |  |  |
|  | **2^-(ΔΔCT values)** |  |  |  |  |
| **Control** | 1 |  |  |  |  |
| **Silibinin** | 16.1112888 |  |  |  |  |
| **Isotrenotinin** | 10.33882265 |  |  |  |  |

**S10 Figure:** The expression of sFRP4 in TNBC MDA-MB-231 cell lines after treatment with silibinin and isotretinoin and its subsequent effects. The cells were subjected to a range of doses of silibinin and isotretinoin, ranging from 12.5 to 50 μM, for 48 hours. The findings of these investigations are presented as the means ± standard error of the mean (SEM) from three separate experiments. Statistical significance was assessed in Figures A, B, and C. The Effect of Silibinin and Isotretinoin affects the mRNA expression of the Wnt/β-catenin antagonist sFRP4 following 48 hours of exposure. Compared to untreated controls, both compounds affect the expression of the sFRP4 gene in Figure C (**P < 0.05, ***P<0.001)

| **Control** | 1 |
| --- | --- |
| **Silibinin** | 16.1112888 |
| **Isotrenotinin** | 10.33882265 |

**Quantitative real-time polymerase chain reaction (qPCR)**

The prepared 10 μL of cDNA Synthesis Mix was added to each RNA/primer mixture, mixed gently, and collected by brief centrifugation. The tube was incubated for 50 min at 50°C. The reaction was terminated at 85°C for 5 min. The cDNA was stored at -20°C. The PCR reaction was performed in a separate tube with gene-specific primers using 2 µl of cDNA templates. The following set of primers was used by Bai et al.,^1^ for real-time PCR. *GAPDH-F*-5’-GGAGTCCCCATCCCAACTCA-3, *GAPDH-R*-5’-GCCCATAACCCCCACAACAC-3’; *SFRP-4-F-5’CCTTTTTGCACTTGCACCGAT*-3’and *SFRP-4-R-5’AGGCAATAGTCACT* CTTCC- 3’. 1 µl (10 µM) of each set of primer, along with 12.5 µl SYBR green PCR master mix (Maxima SYBR Green/ROX qPCR Master Mix (2X)) was used in a total volume of 25 µl. The thermal cycle was set as follows: the first denaturation step was carried out at 95°C for 10 min, followed by 95°C denaturation for 15 sec, the annealing step was carried out at 56°C for 1 min, and the extension step was carried out at 72°C for 1 min. The fold induction was determined by the ΔΔCT method (relative quantification).

1. Bai J, Liu Z, Xu Z, Ke F, Zhang L, Zhu H, et al. Epigenetic downregulation of SFRP4 contributes to epidermal hyperplasia in psoriasis. The Journal of Immunology. 2015;194(9):4185-98.
